# Ensemble Learning with Stochastic Configuration Network for Noisy Optical Fiber Vibration Signal Recognition

**DOI:** 10.3390/s19153293

**Published:** 2019-07-26

**Authors:** Hongquan Qu, Tingliang Feng, Yuan Zhang, Yanping Wang

**Affiliations:** School of Information Science and Technology, North China University of Technology, Beijing 100144, China

**Keywords:** noisy optical fiber vibration signal recognition, optical fiber pre-warning system, stochastic configuration network, bootstrap sampling, AdaBoost

## Abstract

Optical fiber pre-warning systems (OFPS) based on Φ-OTDR are applied to many different scenarios such as oil and gas pipeline protection. The recognition of fiber vibration signals is one of the most important parts of this system. According to the characteristics of small sample set, we choose stochastic configuration network (SCN) for recognition. However, due to the interference of environmental and mechanical noise, the recognition effect of vibration signals will be affected. In order to study the effect of noise on signal recognition performance, we recognize noisy optical fiber vibration signals, which superimposed analog white Gaussian noise, white uniform noise, Rayleigh distributed noise, and exponentially distributed noise. Meanwhile, bootstrap sampling (bagging) and AdaBoost ensemble learning methods are combined with original SCN, and Bootstrap-SCN, AdaBoost-SCN, and AdaBoost-Bootstrap-SCN are proposed and compared for noisy signals recognition. Results show that: (1) the recognition rates of two classifiers combined with AdaBoost are higher than the other two methods over the entire noise range; (2) the recognition for noisy signals of AdaBoost-Bootstrap-SCN is better than other methods in recognition of noisy signals.

## 1. Introduction

At present, inflammable and explosive resources such as oil and natural gas are mainly transported through pipelines, which is convenient and fast. Meanwhile, problems such as resource waste and environmental pollution caused by pipeline leakage also exist. Therefore, real-time monitoring for pipelines’ status is essential [1,2,3]. In the process of pipeline monitoring, the research of OFPS mainly focused on monitoring and recognition of vibration signals [4,5]. In the field of recognition of optical fiber vibration signal, there are two types of institution at present, reflection and interference. The Mach–Zehnder (MZ) method is an interference method, which can be used to detect sensitive characteristics of vibration signal. In addition, the phase-sensitive optical time-domain reflectometer (Φ-OTDR) is a reflection method, which can be used to detect concurrent vibration signals. Compared with other monitoring methods, optical fiber pre-warning systems (OFPS) based on Φ-OTDR are a very common method of long-distance detection and security protection due to their advantage in design (anti-interference, small additional damage, easy upgrade of back-end, low energy consumption in the field and suitable for long-distance defense) [6].

When we choose OFPS based on Φ-OTDR to recognize fiber vibration signals, how to improve the recognition rate of different vibration signals and noisy vibration signals is a challenging task. On the one hand, some scholars studied from the aspect of traditional feature extraction [7,8,9,10,11,12,13,14], on the other hand, some scholars studied from the aspect of combining neural network [15,16], and all of them achieved certain results.

The recognition methods of vibration signals based on neural network is studied by many scholars, because it is simple to operate and does not require too much preprocessing of vibration signals. At the same time, the vibration signal recognition methods based on neural network is also a challenging research field. With the improvement of computing power, neural networks or artificial intelligence will be widely used in the field of optical fiber vibration signal recognition.

The main contribution of this paper is combining original SCN with bootstrap sampling of Bagging and AdaBoost, and proposing three improved methods: Bootstrap-SCN, AdaBoost-SCN, and AdaBoost-Bootstrap-SCN for noisy fiber vibration signal recognition in the case of small sample set. Likewise, the robustness of the proposed methods is tested by using noisy vibration signals. In the experimental part, we first pretreat collected signals and superimpose various types of noise. Then, we send noisy signal sets to five methods (including original SCN and random vector functional link (RVFL) network) for training. At last, we compare the results of five methods.

The article is divided into the following parts: the second section describe the details of related works; the third section briefly introduces original SCN, proposed methods, and methods for generating various types of noisy signals; the fourth section makes a presentation of the optical fiber pre-warning system, original data of fiber vibration signals, and signal pretreating process; the fifth section shows the experiments and analysis; the sixth section is the discussion of experimental results; and the last section concludes this study.

## 2. Related Work

Scholars have done in-depth studies on the recognition technology of optical fiber signals, including instrument improvement, optical fiber signal processing, and classification. Traditional analysis methods are based on time and frequency domain [7,8,9,10]. Bi et al. [11] and Qiu et al. [12] applied constant false alarm rate (CFAR) commonly used in radar signal detection to optical fiber signal processing, and proposed cell averaging-CAFR (CA-CAFR) algorithm with small computational complexity and strong adaptability. However, CA-CAFR algorithm is applicable when the input signals of detector are independent identically distribution (IID) Gaussian random variables, otherwise, ideal effect cannot be achieved. Sun et al. [13] proposed a feature extraction method combining with wavelet energy spectrum and wavelet information entropy, and proved its feasibility in experiments. Yen et al. [14] used wavelet packet transform to extract time domain information from vibration signals to recognize vibration signals, and the experimental results showed that this method could effectively distinguish three kinds of intrusion signals. At the same time, some scholars used neural networks to recognize fiber vibration signals. King et al. [15] designed an optical fiber water sensor system by using signal processing method, neural network and fast Fourier transform (FFT). Makarenko [16] proposed a method for establishing a signal recognition deep learning algorithm in distributed optical fiber monitoring and long period safety systems. It can recognize seven classes of signals and receive time–space data frames as input. However, if a small sample set is used to train deep learning network, the network training will not be in place, which affect recognition effect.

It is also necessary to recognize fiber vibration signals in the case of small sample set. Because not only the acquisition of a large number of fiber vibration signals is difficult, but also the sample calibration work is heavy. Once there is a problem in the calibration of samples, it will directly affect the training of classifier. If a deep network is used for training in the case of small sample set, the network could not be trained effectively. In the field of small networks, Wang et al. [17] proposed an improved classification algorithm that considers multiscale wavelet packet Shannon entropy, which uses radial basis function neural networks for classification and the accuracy rate is 85%. In addition, some new small networks have been created in recent years. Igelnik et al. [18] proposed a three-layer network. Its randomly generated parameters of hidden layer nodes not only improve the generalization performance of network, but also save a lot of iterative operations. Based on RVFL network, Wang et al. [19] proposed stochastic configuration network (SCN) to change the generation method of hidden layer nodes, which makes the size setting of network more flexible.

At the same time, since OFPS usually works in a complex environment and is susceptible to various disturbances, the acquired fiber vibration signals contain various noises. During the propagation of backscattered light, the remaining types of scattered light generated in fiber may become the optical noise of the system. Photoelectric detectors based on avalanche photo diode module (APD) generate random noise during the fiber conversion and amplification process. All these noises overwhelm the fiber signals of the system [20]. Sun et al. [21] used the theoretical model to verify the influence of Rayleigh optical noise more intuitively on the accuracy of system measurements. It can be seen that various types of noises seriously affect the recognition of fiber vibration signals. Therefore, it is an important research field to improve the recognition accuracy of fiber vibration signals in noisy environment.

For the recognition task of noisy fiber signals, common methods are to use synchronization overlapping average algorithm to suppress the white noise of the system. The increase of the number of accumulations can effectively promote the signal–noise ratio (SNR) and improve the performance of the system [22]. Since the structure of self-noise is similar to random noise, and median filter is an effective method to remove random noise, Qin et al. [23] moved the noise from signals in three-dimensional time domain with median filter. In order to eliminate the influence of coherent Rayleigh noise in coherent optical time domain reflectometry (COTDR) systems, Liang et al. [24] proposed a noise reduction method based on timed frequency hopping. This method could effectively suppress the noise in COTDR system and improve the long-distance sensing performance of the system. In order to reduce the influence of background noise on vibration detection, Ibrahim Ölçer et al. [25] proposed an adaptive time-matching filtering method, which significantly improved the SNR of independent adaptive processing. However, when performing long-distance monitoring, it is necessary to introduce optical amplification to increase the distance of laser irradiation, which will affect the effect of this method.

In the recognition of noisy fiber vibration signals with a small sample set, the problem of low classification accuracy still occurs in the use of SCN, and the recognition rate is sometimes even lower than 50%. This shows that the SCN after training is less robust in this case. Combining models with ensemble learning methods is a common choice in solving the problem of low robustness [26,27]. In the field of ensemble learning methods, Boosting and Bagging are two representative types of methods. Fernandes et al. [28] used AdaBoost and random weight neural networks (RWNN) in the spectral data processing of grape stems to obtain better classification results than other methods. Asim et al. [29] also proposed an earthquake prediction system that combines earthquake prediction indicators with genetic programming and AdaBoost, and obtained better results in earthquake prediction. Likewise, Okujeni et al. [30] used a small share of training samples and a low number of Bagging iterations to generate accurate urban fraction maps. Akila et al. [31] combined the Bagging method with Risk Induced Bayesian Inference method to form a cost-sensitive weighted voting combiner, which indicated 1.04–1.5 times reduced cost in the Brazilian bank data experiment. Wing et al. [32] proposed a bagging boosting-based semi-supervised multi-hashing method with query-adaptive re-ranking, which outperformed state-of-the-art hashing methods with statistical significance.

## 3. Theoretical Explanation

### 3.1. Original SCN

**Figure 1:**
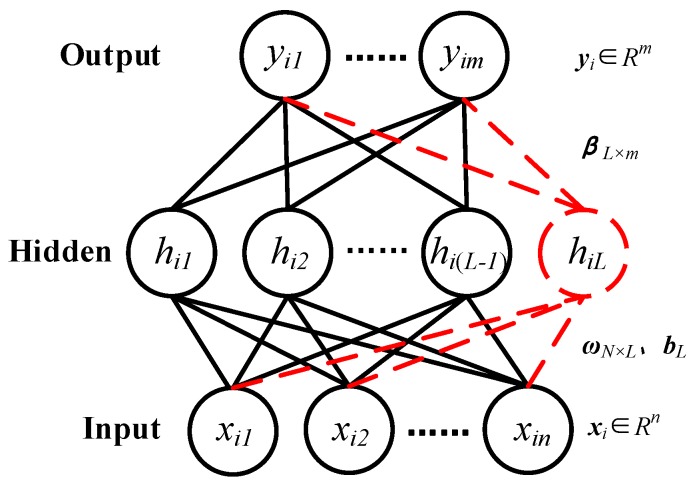
The structure of SCN network [18].

SCNs are a class of randomized neural networks proposed by Wang and Li in [18], which originally contributed to the development of randomized learning techniques. Its structure is shown in Figure 1.

From Figure 1, we can see that the input is $X_{n\times N}=\left[x_{1},\cdots ,x_{i},\cdots ,x_{N}\right]$, $x_{i}={\left[x_{i1},\cdots ,x_{ij},\cdots ,x_{in}\right]}^{T}\in R^{n}$ and the output is $Y_{m\times N}=\left[y_{1},\cdots ,y_{i},\cdots ,y_{N}\right]$, $y_{i}={\left[y_{i1},\cdots ,y_{ij},\cdots ,y_{im}\right]}^{T}\in R^{m}$, where *N* is the number of training samples, *n* is the dimension of sample’s input, *m* is the dimension of sample’s output. Meanwhile, we define the input weight matrix as $\omega_{n\times L}=\left[\omega_{1},\cdots ,\omega_{i},\cdots ,\omega_{L}\right]\in R^{n\times L}$, the bias vector as $b_{L}={\left[b_{1},\cdots ,b_{i},\cdots ,b_{L}\right]}^{T}\in R^{L}$, where *L* is the number of hidden layer nodes. Then, we calculate the output of hidden layer nodes of network according to the input of network, input weight matrix and bias vector. Herein, the output of the *L*th hidden layer node is expressed as

(1) $$h_{L}\left(X\right)={\left[h_{1L},\cdots ,h_{iL},\cdots ,h_{NL}\right]}^{T}=\sigma \left(\omega_{L}^{T}X+b_{L}\right),$$

where

(2) $$h_{iL}=\sigma \left(\omega_{L}^{T}x_{i}+b_{L}\right)=\left[\frac{1}{1+e^{-\omega_{L}^{T}x_{i}+b_{L}}}\right].$$

The output weight matrix is $\beta_{L\times m}=\left[\beta_{1},\cdots ,\beta_{i},\cdots ,\beta_{m}\right]$, $\beta_{i}={\left[\beta_{i1},\cdots ,\beta_{ij},\cdots ,\beta_{iL}\right]}^{L}\in R^{L}$. Combine with the output of hidden layer $H\left(X\right)=\left[h_{1}\left(X\right),\cdots ,h_{i}\left(X\right),\cdots ,h_{L}\left(X\right)\right]$ and the weight matrix of output $\beta_{L\times m}$, we can get corresponding output $y_{i}^{\prime }=[y_{i1}^{\prime },\cdots ,y_{ij}^{\prime },\cdots ,y_{im}^{\prime }]\in R^{m}$. Meanwhile, we can calculate the current corresponding residual matrix $E_{N\times m}\left(X\right)$ of network according to the output $y_{i}$ corresponded to the input

(3) $$E_{N\times m}\left(X\right)=\left[e_{\left(l-1\right)1}\left(X\right),\cdots ,e_{\left(l-1\right)j}\left(X\right),\cdots ,e_{\left(l-1\right)m}\left(X\right)\right],$$

where

(4) $$e_{\left(l-1\right)j}\left(X\right)={\left[e_{(l-1)j}\left(x_{1}\right),\cdots ,e_{(l-1)j}\left(x_{i}\right),\cdots ,e_{(l-1)j}\left(x_{N}\right)\right]}^{T}\in R^{N},$$

(5) $$e_{(l-1)j}\left(x_{i}\right)=\left|y_{ij}-y_{ij}^{\prime }\right|.$$

When adding the *L*th hidden layer node, the constraint condition is expressed as

(6) $$\xi_{Lj}=\frac{{\left(e_{(L-1)j}^{T}\left(X\right)\cdot h_{L}\left(X\right)\right)}^{2}}{h_{L}^{T}\left(X\right)\cdot h_{L}\left(X\right)}-\left(1-r-\mu_{L}\right)e_{(L-1)j}^{T}\left(X\right)e_{(L-1)j}\left(X\right)>0,$$

where j=1,2,⋯,m, *r* is a sequence greater than 0 and less than 1, and varies in the process of finding parameters, $\mu_{L}\leq 1-r$ and $\underset{L\to \infty }{\lim}\mu_{L}=0$.

The training process of SCN is as follows. When adding the *L*th hidden layer node, $\omega_{iL}$ and $b_{\mathrm{iL}}$ are randomly generated within a certain range ${\left[\lambda_{\min},\lambda_{\max}\right]}^{n}$ and $\left[\lambda_{\min},\lambda_{\max}\right]$, and made up into a candidate set $\left\{\left(\omega_{1L},b_{1L}\right),\cdots ,\left(\omega_{iL},b_{\mathrm{iL}}\right),\cdots \left(\omega_{uL},b_{uL}\right)\right\}$, where *u* is the number of candidate nodes. Then, one set $\left(\omega_{iL},b_{iL}\right)$ is selected when $\xi_{Lj}>0$ (6) and minimizing $\varepsilon =1/N\sum_{i=1}^{N}\left|y_{i}-y_{i}^{\prime }\right|$. If there is no one in the candidate set satisfies the constraint condition, the range will be appropriately expanded and the selection will be made again, but the scope of scalability is limited. The network stops training when it meets one of the following three conditions: (1) the number of hidden layer nodes reaches to the preset maximum value $L_{max}$; (2) the training error is lower than the preset error value $\varepsilon_{min}$; (3) the $\left(\omega_{iL},b_{iL}\right)$ that satisfies the condition cannot be found within the maximum range.

It can be seen from the above description that the difference between SCN and other neural networks lies in its unique training mode. It gives up the traditional iterative method of updating network parameters, and uses the pattern of adding hidden layer nodes under constraints, which makes the scale of network flexible and controllable, and greatly reduces the loss of computing resources.

### 3.2. SCN-Based Methods

The robustness of the model refers to the ability to resist or overcome adverse conditions. In these experiments, the stronger models’ ability to overcome noisy environment, the better the robustness of models. In order to strengthen the robustness of original SCN, we combine the original SCN with two ensemble learning methods. We also strengthen the robustness of model by combining two ensemble learning methods based on original SCN.

#### 3.2.1. Bootstrap-SCN Method

Bagging is a parallel ensemble learning method, its process is as follows. First, we randomly sample a number of different training subsets with the same number of training set samples from training set, and this step is called bootstrap sampling. Then, we use these different training subsets to train corresponding base classifiers with the same type of classifier models. Finally, these base classifiers are combined by simple voting or other methods to form the final classifier. Here, bootstrap sampling is the core step of bagging.

By using bootstrap sampling in the case of small training set, on the one hand, these base classifiers will have a larger difference between each other according to training subsets; On the other hand, it also avoids the problem that training subsets are too small which caused by dividing the completely different subsets, and finally the base classifiers are not trained well.

**Figure 2:**
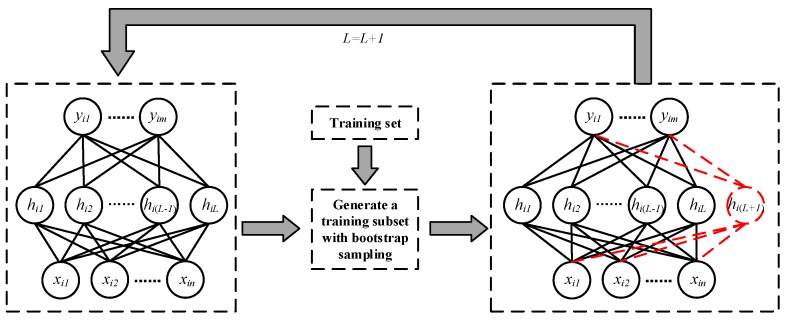
The flow of Bootstrap-SCN method.

Combined with the training process of SCN, we propose Bootstrap-SCN method. This method generates a training subset by using bootstrap sampling before adding hidden layer nodes to SCN. This training subset is used for the generation of new hidden layer node of SCN. After that, we repeat this step to generate more hidden layer nodes until the end of training. The flow of this method is shown in Figure 2, and the pseudo code is shown in Algorithm 1. 


**Algorithm 1: Pseudo code of Bootstrap-SCN method.**
Given training set $T=\left\{\left(x_{1},y_{1}\right),\cdots ,\left(x_{i},y_{i}\right),\cdots ,\left(x_{N},y_{N}\right)\right\}$, training subset by bootstrap sampling $\left\{T_{1},\cdots ,T_{j},\cdots T_{n}\right\}$, SCN model Γ, current and maximum number of hidden layer nodes *L* and *L_max_*, training error ε, expected error tolerance $\varepsilon_{\min}$
Initialize Γ and ε;1: **While**
$L\leq L_{max}$ AND $\varepsilon >\varepsilon_{min}$ do2:   Generate $T_{j}$ using bootstrap sampling on the basis of T;3:   Train Γ and generate a new hidden layer node with $T_{j}$;4:   j=j+1;5: **End while****Return:**G(x)=Γ(T).

#### 3.2.2. AdaBoost-SCN Method

When we train network with small sample sets, it is very likely that the network training is not in place and the robustness of network is poor. AdaBoost can be used to reduce the deviation and improve the effect of network on the vibration signal recognition [33]. Meanwhile, because the hidden layer nodes of original SCN are generated one after another, which conforms to the form of serially training different base classifiers of AdaBoost. In this method, we use AdaBoost when generating the hidden layer nodes of SCN, so as to improve the effect of original SCN in fiber vibration signal recognition.

**Figure 3:**
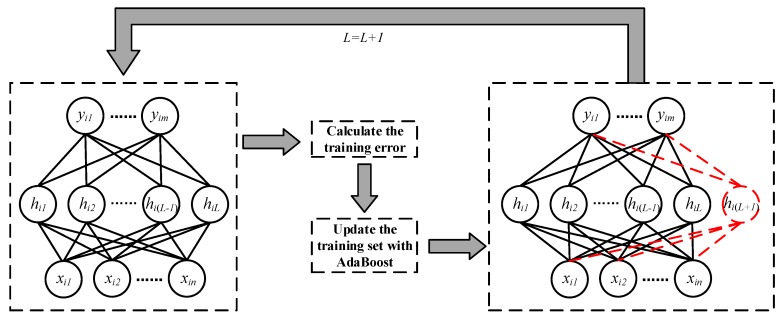
The flow of AdaBoost-SCN method.

In the process of SCN training, we consider the hidden layer nodes generated by each training round as different classifiers. When SCN is in the initial state, the weights of samples are the same. When training the network, the samples’ weights are adjusted according to the training error of SCN and the recognition results of samples after each time the hidden layer node is added, and are used for the calculation of training error when the hidden layer node is added next time. Then, we repeat this step to generate more hidden layer nodes until the end of training. The flow of this method is shown in Figure 3, and the pseudo code is shown in Algorithm 2. For the sake of simplicity, we do not assign weights to the hidden layer nodes of trained network, which means all hidden layer nodes have the same weights in calculation process.


**Algorithm 2: The pseudo code of AdaBoost-SCN method.**
Given training set $T=\left\{\left(x_{1},y_{1}\right),\cdots ,\left(x_{i},y_{i}\right),\cdots ,\left(x_{N},y_{N}\right)\right\}$, SCN model Γ, current and maximum number of hidden layer nodes *L* and *L_max_*, training error, expected error tolerance $\varepsilon_{min}$, weights of training set $D_{k}=\left(\alpha_{k1},\cdots ,\alpha_{ki},\cdots ,\alpha_{kN}\right)$
Initialize Γ and ε, $D_{1}\left(x\right)=1/N$, k=1;1: **While**
$L\leq L_{max}$ AND $\varepsilon >\varepsilon_{min}$ Do2:   $G\left(x\right)=\Gamma \left(T,D_{k}\right)$;3:   $\varepsilon =P_{x∼D_{k}}\left(G\left(x\right)\neq y\right)$;4:   if ε>1/2 then set L=L−1 and abort loop5:   $\delta =\frac{\varepsilon }{1-\varepsilon }$;6:   $D_{k+1}\left(x\right)=\{\begin{array}{ll} D_{k}\left(x\right)\delta^{1-I\left(G\left(x_{i}\right)\neq y_{i}\right)}, & \mathrm{if}\;G\left(x\right)=f\left(x\right)\\ D_{k}\left(x\right), & \mathrm{if}\;G\left(x\right)\neq f\left(x\right) \end{array}$;7: **End while****Return**G(x)=Γ(T).

#### 3.2.3. AdaBoost-Bootstrap-SCN Method

According to Section 3.2.1 and Section 3.2.2, the two main ensemble learning methods can be used in the training of SCN and applied to the recognition of fiber vibration signals. From the perspective of bias-variance decomposition, AdaBoost mainly focuses on reducing the bias of classifier, while bagging mainly focuses on reducing the variance of classifier. However, there is a conflict between bias and variance in the training process of a classifier. When the classifier is not trained enough, it cannot fit neither training set and testing set well. At this time, the bias of the classifier dominates the classification error rate. As the training continues, when the training is too much, the classifier can fit training set well, but it cannot fit testing set well. At this time, the variance dominates the classification error rate [34]. Therefore, in order to balance the bias and variance of classifier, we could combine these two ensemble learning methods with SCN simultaneously.

**Figure 4:**
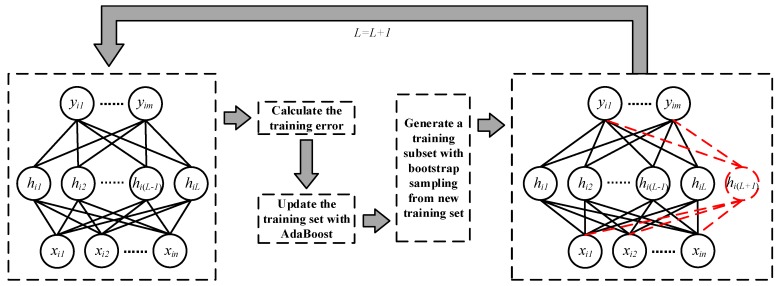
The flow of AdaBoost-Bootstrap-SCN method.

The way we combine two ensemble learning methods with SCN is as follows. During the process of training SCN, we need to update the weights of the samples according to the training error and samples’ recognition results of current network before adding new hidden layer node. Then we use bootstrap sampling to generate a training subset and choose the corresponding weights of samples in the training subset. At last, we use this training subset and the corresponding weights to train SCN and obtain a new hidden layer node. We repeat this step to generate more hidden layer nodes until the end of training. The flow of this method is shown in Figure 4, and the pseudo code is shown in Algorithm 3. For the sake of simplicity, we do not assign weights to the hidden layer nodes of the trained network, which means all hidden layer nodes have the same weights in calculation process.


**Algorithm 3: The pseudo code of AdaBoost-Bootstrap-SCN method.**
Initialize Γ and ε, $D_{1}\left(x\right)=1/N$, k=1;1: **While**
$L\leq L_{max}$ AND $\varepsilon >\varepsilon_{min}$ do2:   Generate $T_{j}$ using Bootstrap Sampling on the basis of T;3:   Generate $D_{jk}$ with $D_{k}$ and $T_{j}$;4:   $G\left(x\right)=\Gamma \left(T_{j},D_{jk}\right)$;5:   $\varepsilon =P_{x∼D_{jk}}\left(G\left(x\right)\neq y\right)$;6:   if ε>1/2 then set L=L−1 and abort loop7:   $\delta =\frac{\varepsilon }{1-\varepsilon }$;8:   ${D^{\prime }}_{jk}\left(x\right)=\{\begin{array}{ll} D_{jk}\left(x\right)\delta^{1-I\left(G\left(x_{i}\right)\neq y_{i}\right)}, & \mathrm{if}\;G\left(x\right)=f\left(x\right)\\ D_{jk}\left(x\right), & \mathrm{if}\;G\left(x\right)\neq f\left(x\right) \end{array}$;9:   Update $D_{k}$ with ${D^{\prime }}_{jk}$ to $D_{k+1}$;10: **End while****Return**G(x)=Γ(T).

### 3.3. Simulated Noise

Due to the unique nature of OFPS, it needs to work in complex environments for long periods of time. The various noises also cause some interference to the recognition of the vibration signals of system. In order to eliminate the influence of noises, we need to improve the robustness of classifiers. In this paper, we mainly use four common noises for simulation and verify the effectiveness of the proposed methods.

#### 3.3.1. White Gaussian Noise

White Gaussian noise is a kind of noise whose instantaneous value obeys Gaussian distribution and power spectral density obeys uniform distribution. It is commonly used to simulate ambient noise because its probability distribution is normally distributed. In order to analyze the influence of noise on signal recognition performance, we combine pretreated fiber vibration signals with white Gaussian noise to obtain noisy vibration signals

(7) $$P_{i}^{G}\left(t\right)=P_{i}\left(t\right)+\alpha_{G}\cdot n_{i}^{G},$$

where $P_{i}\left(t\right)$ is original fiber vibration signal, $\alpha_{G}$ is the multiple of superimposed noise, $P_{i}^{G}\left(t\right)$ is the fiber vibration signal superimposed by Gaussian white noise, $n_{i}^{G}∼N\left(0,1\right)$ is simulated white Gaussian noise. The frequency domain expression of $n_{i}^{G}$ is

(8) $$N_{i}^{G}\left(z\right)=\frac{1}{\sqrt{2\pi }}exp\left(-\frac{z^{2}}{2}\right).$$

In order to accurately understand the interference of different noise intensities on signal recognition, we use the variable $\alpha_{G}$ to control the amplitude of noise, and the same operation is applied to the subsequent noise.

#### 3.3.2. White Uniform Noise

White uniform noise refers to the noise whose power spectral density is constant in the whole frequency domain. In this paper, the vibration signal superimposed white uniform noise is defined as $P_{i}^{U}$

(9) $$P_{i}^{U}\left(t\right)=P_{i}\left(t\right)+\alpha_{U}\cdot n_{i}^{U},$$

where $P_{i}\left(t\right)$ is original fiber vibration signal, $\alpha_{U}$ is the multiple of superimposed noise, $P_{i}^{U}\left(t\right)$ is the fiber vibration signal superimposed by white uniform noise. The frequency domain expression of $n_{i}^{U}$ is

(10) $$N_{i}^{U}\left(z\right)=\{\begin{array}{c} \frac{1}{b-a}\;,\;a\leq z\leq b\\ 0\;,\;others \end{array}.$$

In this paper, the parameters of white uniform noise are set to: a=−5,b=5.

#### 3.3.3. Raleigh Distributed Noise

Rayleigh distributed noise refers to the noise whose instantaneous value obeys Rayleigh distribution. In this paper, the vibration signal superimposed Raleigh distributed noise is defined as $P_{i}^{R}$

(11) $$P_{i}^{R}\left(t\right)=P_{i}\left(t\right)+\alpha_{R}\cdot n_{i}^{R},$$

where $P_{i}\left(t\right)$ is original fiber vibration signal, $\alpha_{R}$ is the multiple of superimposed noise, $P_{i}^{R}\left(t\right)$ is the fiber vibration signal superimposed by Raleigh distributed noise. The frequency domain expression of $n_{i}^{R}$ is

(12) $$N_{i}^{R}\left(z\right)=\frac{z}{\sigma^{2}}\exp\left(-\frac{z^{2}}{2\sigma^{2}}\right),z\geq 0.$$

In this paper, the parameter of Raleigh distributed noise is set to: σ=2.

#### 3.3.4. Exponentially Distributed Noise

Exponentially distributed noise refers to the noise whose instantaneous value obeys exponential distribution. In this paper, the vibration signal superimposed exponentially distributed noise is defined as $P_{i}^{E}$

(13) $$P_{i}^{E}\left(t\right)=P_{i}\left(t\right)+\alpha_{E}\cdot n_{i}^{E},$$

where $P_{i}\left(t\right)$ is original fiber vibration signal, $\alpha_{E}$ is the multiple of superimposed noise, $P_{i}^{E}\left(t\right)$ is the fiber vibration signal superimposed by exponentially distributed noise. The frequency domain expression of $n_{i}^{E}$ is

(14) $$N_{i}^{E}\left(z\right)=\{\begin{array}{ll} \lambda e^{-\lambda z} & ,z>0\\ 0 & ,z\leq 0 \end{array}.$$

In this paper, the parameter of exponentially distributed noise is set to: λ=2.5.

## 4. Description of System and Data

### 4.1. Optical Fiber Pre-Warning System (OFPS)

**Figure 5:**
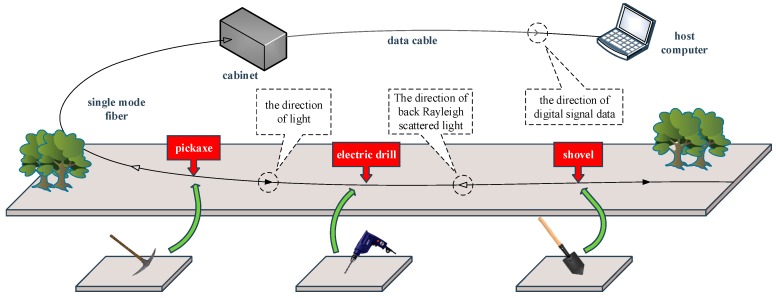
The schematic diagram of the OFPS.

The acquisition of fiber vibration signals was completed by the OFPS developed by the authors’ laboratory, and the schematic diagram of the OFPS is shown in Figure 5. The OFPS uses Φ-OTDR to detect intrusion vibration signals. Specifically, light is emitted from the cabinet and injected into the single mode fiber. Vibrations generated on the ground change the refractive index of fiber in that place, causing the back Rayleigh scattered light to change. After the acquisition, we analyze the Rayleigh scattered light and judge the type of vibration generated on the ground according to the change.

### 4.2. Fiber Vibration Data and Sample Pretreating

**Figure 6:**
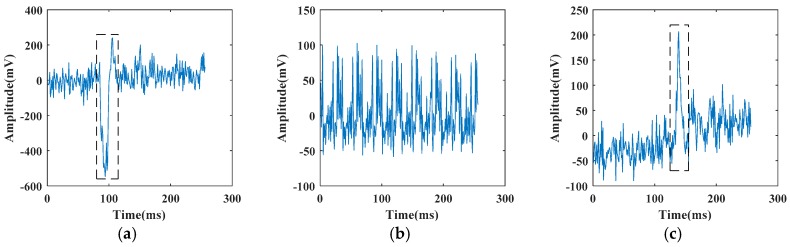
Fiber vibration signals in time domain: (**a**) pickaxe; (**b**) electric drill; (**c**) shovel.

The fiber vibration signals used in this experiment was collected from the experimental site in the suburbs of Beijing. These signals include pickaxe, electric drill, and shovel. When we perform pretreating process on these signals, we first remove the direct current (DC) part of the acquired signals. Three types of vibration samples after removing DC part are shown in Figure 6. As it can be seen from the figures, three signals have different characteristics. Wherein, the electric drill signal (b) has periodicity, and the amplitude of the pickaxe signal (a) is greater than the shovel signal (c).

**Figure 7:**
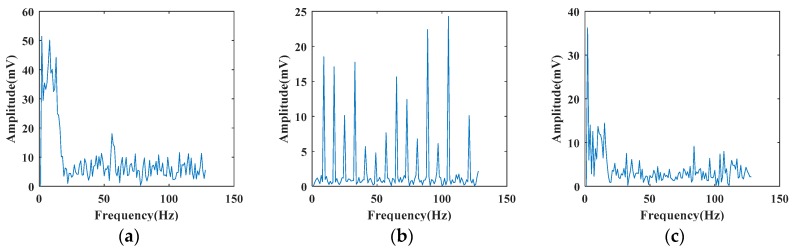
Fiber vibration signals in frequency domain: (**a**) pickaxe; (**b**) electric drill; (**c**) shovel.

Meanwhile, the dashed boxes in Figure 6a,c show that the vibration part of a signal has a short duration and the position in the time dimension is not fixed, which makes the vibration parts of the same type of signals have different position in the time dimension. If the samples are sent directly to the classifier for training after removing DC part, it will affect the recognition of classifier. FFT transforms the signal from time domain to frequency domain, which ignores the information of time domain, and unifies the vibration signals of same type. Therefore, we perform 128-point FFT on vibration signals. Figure 7 shows the result of performing FFT on the signals shown in Figure 6.

Then we randomly divide these three kinds of signals into a training set and a testing set in a 4:1 ratio. The training set is a set of samples used for learning, which is to fit the parameters of the classifier. The testing set is used only to assess the performance of a fully specified classifier. The data is shown in Table 1.

**Figure 8:**
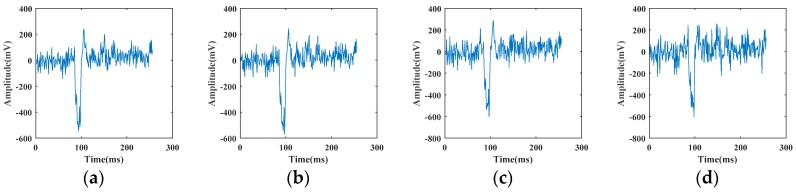

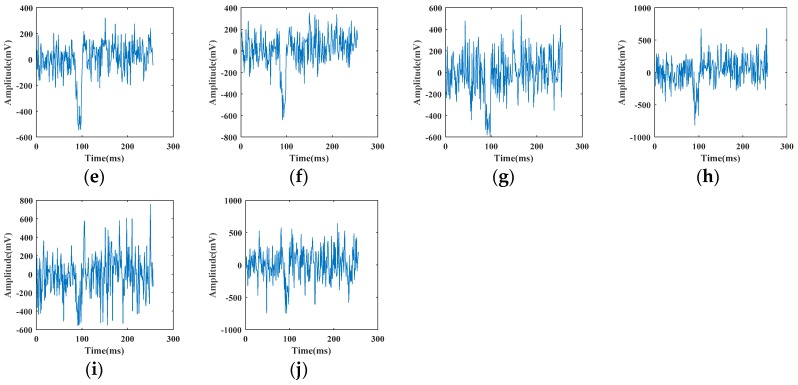
Pickaxe signal superimposed with white Gaussian noise according to Equation (9): (**a**) $\alpha_{G}=20$; (**b**) $\alpha_{G}=40$; (**c**) $\alpha_{G}=60$; (**d**) $\alpha_{G}=80$; (**e**) $\alpha_{G}=100$; (**f**) $\alpha_{G}=120$; (**g**) $\alpha_{G}=140$; (**h**) $\alpha_{G}=160$; (**i**) $\alpha_{G}=180$; (**j**) $\alpha_{G}=200$.

In our experiments, we assume that the vibration signals used are relatively pure and simulate the noisy signal recognition task by superimposing different artificial noises. It can be seen from Figure 6 that the largest amplitude of vibration signals is about 800 mV in pickaxe signal (a). In order to fully analyze various degrees of noisy interference, we add integer multiples of various noises according to Equations (7), (9), (11), and (13) on the basis of original vibration signals until it completely covers all signals. Figure 8 shows the results of superimposing different multiples of white Gaussian noise based on pickaxe signal in Figure 6a.

## 5. Numerical Experiment

After preparing fiber vibration signals and the signal sets superimposed by four different noises, we use these noisy signal sets to train SCNs which combine with different ensemble learning methods. The recognition effects of different methods on the noisy signal sets is obtained and compared. In order to eliminate the influence of the random generation of hidden layer nodes on the experimental results, we repeat each experiment and average the experimental results. Here, we set network to stop training when the hidden layer nodes of network exceed 100. Meanwhile, the maximum number of hidden layer candidate nodes is set to 100 (one of these candidate nodes is selected when adding a hidden layer node). During the training process, each time a hidden layer node is added, the accuracy of training and testing is recorded.

**Figure 9:**
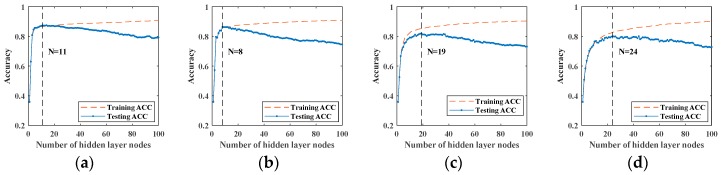

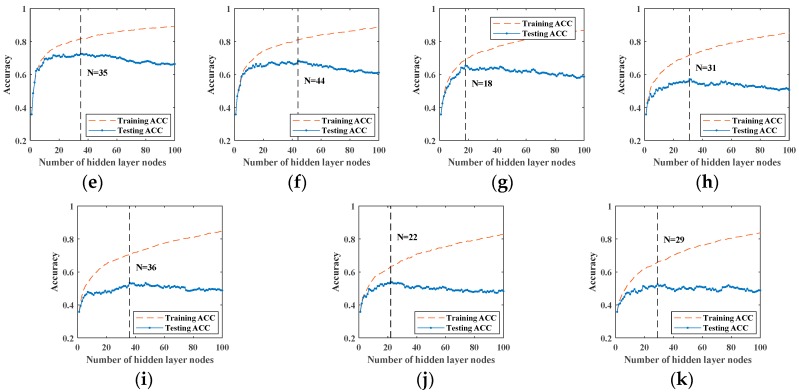
Original SCN’s training and testing accuracy for signals superimposed by white Gaussian noise: (**a**) $\alpha_{G}=0$; (**b**) $\alpha_{G}=20$; (**c**) $\alpha_{G}=40$; (**d**) $\alpha_{G}=60$; (**e**) $\alpha_{G}=80$; (**f**) $\alpha_{G}=100$; (**g**) $\alpha_{G}=120$; (**h**) $\alpha_{G}=140$; (**i**) $\alpha_{G}=160$; (**j**) $\alpha_{G}=180$; (**k**) $\alpha_{G}=200$.

**Figure 10:**
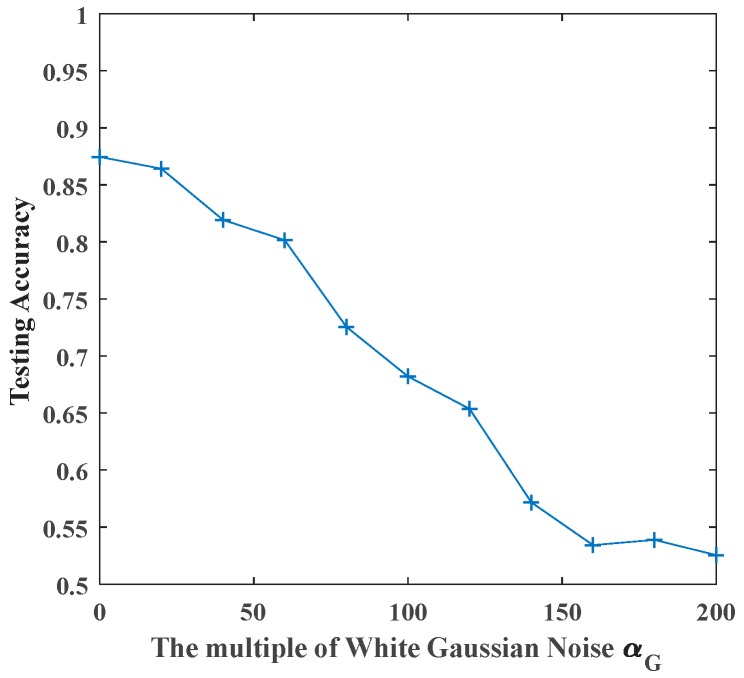
The maximum testing accuracy of original SCN with white Gaussian noise.

Here, we have four classifiers including original SCN, Bootstrap-SCN, AdaBoost-SCN, and AdaBoost-Bootstrap-SCN and different multiples of white Gaussian noise, white uniform noise, Rayleigh distributed noise and exponentially distributed noise. First, we train four different types of classifiers with the signal sets superimposed by different types and intensities of noises. In order to eliminate the influence of randomness on experimental results, each sample set is performed 10 times of training and testing on each classifier. Then, the experimental results are averaged to obtain the actual classification effect of classifier in current sample set. Afterwards, we find the position with the highest testing accuracy in the case of each intensity of noises, and use the highest testing accuracy to form the testing accuracy curve. In addition, we use this training method to obtain the results for other signal sets superimposed by different types and intensities of noises. Figure 9 shows the average results when signals superimposed by different intensities of white Gaussian noise are sent to original SCN. The positions of the maximum testing accuracy are also indicated in figures. Meanwhile, the testing accuracy curve composed of the highest testing accuracy is shown in Figure 10.

### 5.1. Recognition with Original SCN

**Figure 11:**
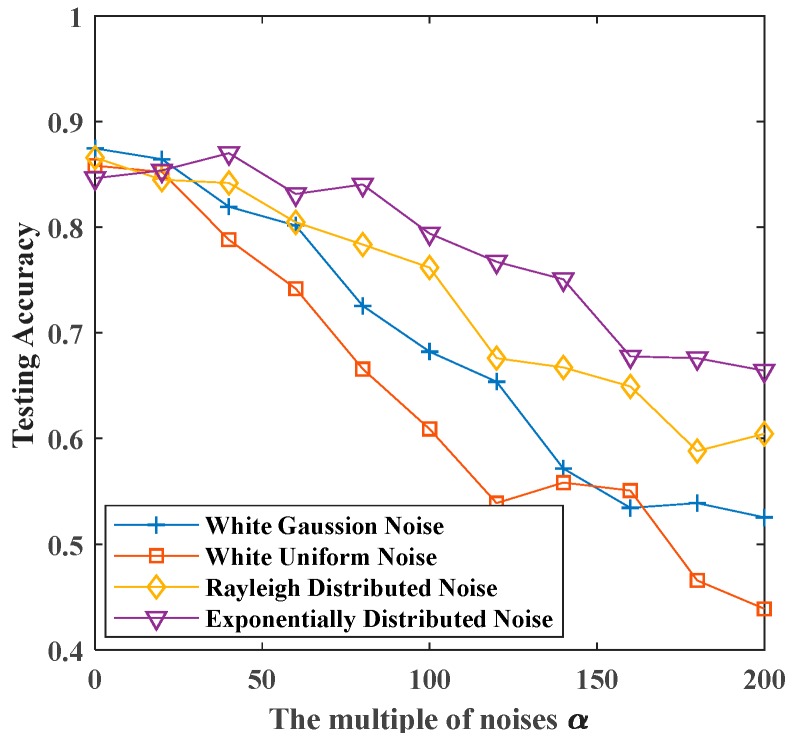
The maximum testing accuracy of original SCN with different noises.

According to the calculation method mentioned above, we first use original SCN to recognize the vibration signals superimposed by four kinds of noises. The results are shown in Figure 11.

From Figure 11, we can see that the recognition accuracy of original SCN decreases with the increase of noise’s amplitude. By observing the recognition effect of signals with equal multiple of noises, it can be seen that different noises have different degrees of interference with signals. For white Gaussian noise, testing accuracy decreases from 0.8746 of noiseless signals to 0.5254 when the multiple of noises is 200; For white uniform noise, the testing accuracy decreases from 0.8582 of noiseless signals to 0.4388 when the multiple of noises is 200. For Rayleigh distributed noise and exponentially distributed noise, the testing accuracy decreases from 0.8657 and 0.8463 with the noiseless signal to 0.6045 and 0.6642 when the multiple of noises is 200.

### 5.2. Recognition with Bootstrap-SCN

**Figure 12:**
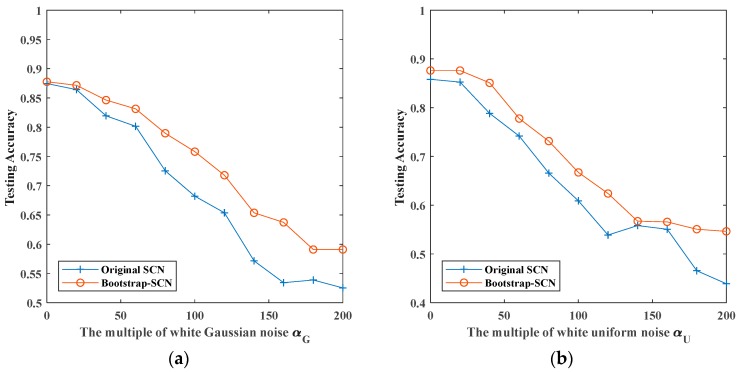

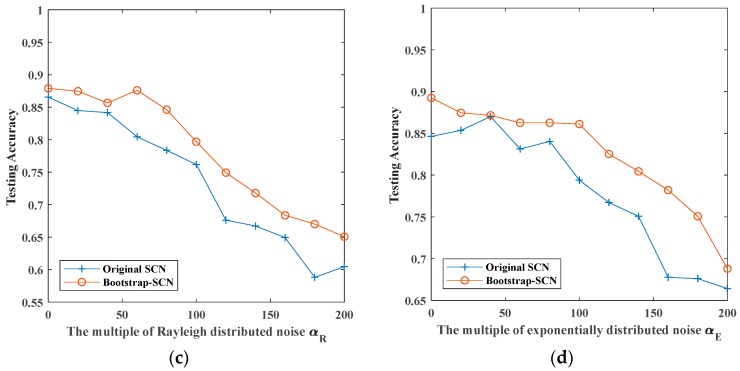
The maximum testing accuracy of original SCN and Bootstrap-SCN with different noises: (**a**) white Gaussian noise; (**b**) white uniform noise; (**c**) Rayleigh distributed noise; (**d**) exponentially distributed noise.

In this section, we combine bootstrap sampling with SCN to recognize noisy vibration signals. The comparison of the recognition accuracy between original SCN and Bootstrap-SCN is shown in Figure 12.

Figure 12 shows the recognition rates of original SCN and Bootstrap-SCN as the superimposed noises’ multiplier increases. When the multiplier of superimposed noise increases, the recognition rates of both methods decrease. For signals superimposed by white Gaussian noise, the recognition rate of Bootstrap-SCN decreased from 0.8776 of noiseless signals to 0.5910 when the multiple of noises is 200. For signals superimposed by white uniform noise, Rayleigh distributed noise and exponentially distributed noise, the recognition rate of Bootstrap-SCN decreased from 0.8761, 0.8791, and 0.8925 of noiseless signals to 0.5463, 0.6507, and 0.6881 when the multiple of noises is 200.

The above results show that Bootstrap-SCN and original SCN have the same recognition effect when there is no superimposed noise. In the case of superimposing four kinds of noises, the recognition rate of Bootstrap-SCN is slightly higher than that of original SCN, but the effect is not obvious.

### 5.3. Recognition with AdaBoost-SCN

**Figure 13:**
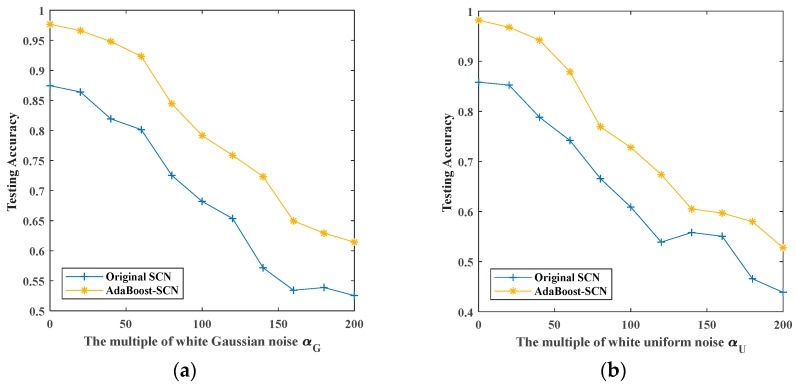

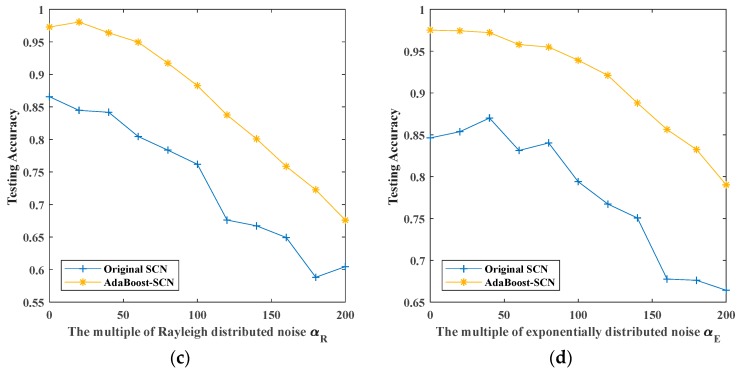
The maximum testing accuracy of original SCN and AdaBoost-SCN with different noises: (**a**) white Gaussian noise; (**b**) white uniform noise; (**c**) Rayleigh distributed noise; (**d**) exponentially distributed noise.

In this part, we use another ensemble learning method to improve the recognition rate of SCN. We combine AdaBoost with SCN to recognize four kinds of noisy signals. The recognition results of noisy signals by original SCN and AdaBoost-SCN are shown in Figure 13.

Figure 13 shows the recognition rates of four kinds of noisy signals by original SCN and AdaBoost-SCN as the superimposed noises’ multiplier increases. For signals superimposed by white Gaussian noise and white uniform noise, the recognition rate of AdaBoost-SCN decreases from 0.9767 and 0.9820 of noiseless signals to 0.6143 and 0.5278 when the multiple of noises is 200. For signals superimposed by Rayleigh distributed noise and exponential distributed noise, the recognition rate of AdaBoost-SCN decreases from 0.9729 and 0.9752 of noiseless signals to 0.6759 and 0.7902 when the multiple of noises is 200. It can be seen from the comparison with original SCN that the recognition accuracy of AdaBoost-SCN is about 0.1 higher than that of original SCN in the case of different superimposed noise.

### 5.4. Recognition with AdaBoost-Bootstrap-SCN

**Figure 14:**
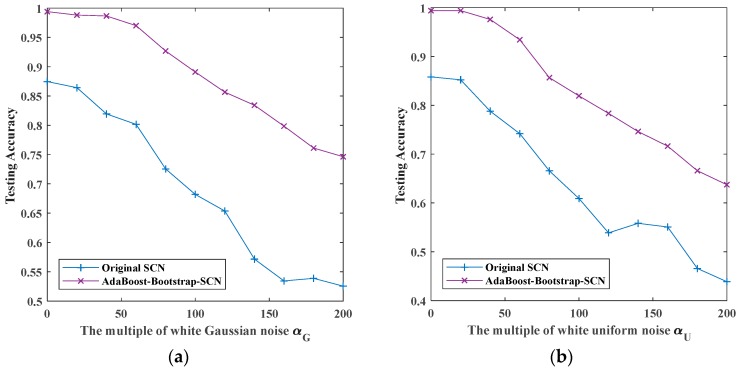

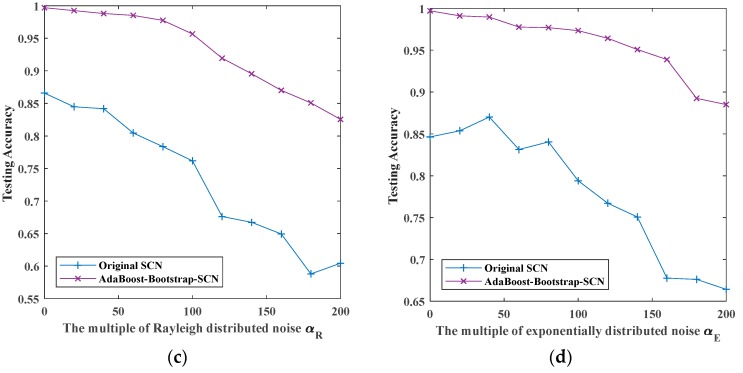
The maximum testing accuracy of original SCN and AdaBoost-Bootstrap-SCN with different noises: (**a**) white Gaussian noise; (**b**) white uniform noise; (**c**) Rayleigh distributed noise; (**d**) exponentially distributed noise.

After the above experiments of two combination methods, we carried out experimental verification on the proposed AdaBoost-Bootstrap-SCN. The recognition rates of noisy signals by original SCN and AdaBoost-Bootstrap-SCN are shown in Figure 14.

Figure 14 shows the recognition rates of four kinds of noisy signals by original SCN and AdaBoost-Bootstrap-SCN as the superimposed noises’ multiplier increases. For signals superimposed by white Gaussian noise and white uniform noise, the recognition rates of AdaBoost-Bootstrap-SCN decreased from 0.9940 and 0.9940 of noiseless signals to 0.7463 and 0.6373 when the multiple of noises is 200. For signals superimposed by Rayleigh distributed noise and exponential distributed noise, the recognition rate of AdaBoost-Bootstrap-SCN decreased from 0.9970 and 0.9970 of noiseless signals to 0.8254 and 0.8850 when the multiple of noises is 200. Compared with original SCN, AdaBoost-Bootstrap-SCN can improve 0.1–0.15 on the basis of original SCN in the case of no noise superposition, and the recognition rate is more than 0.99. Meanwhile, the recognition of this method is improved more than 0.2 on the basis of original SCN when the multiple of noises is 200.

### 5.5. Comparison with Other Methods

Because the structure of RVFL network is similar to SCN, we use RVFL network for comparison. RVFL network have three layers. Its input layer has 128 nodes, which is the same as the dimension of vibration samples. The number of output layer nodes is the same as the dimension of sample’s label. As the number of hidden layer nodes is a hyper-parameter, which need be set before network training, we need to record the experimental results of RVFL network with different numbers of hidden nodes. In order to eliminate the influence of the randomness in network training, we repeat each experiment 10 times and average experimental results. The condition of stopping training is set as: when testing error is less than 0.01. The experimental results are as follows.

**Figure 15:**
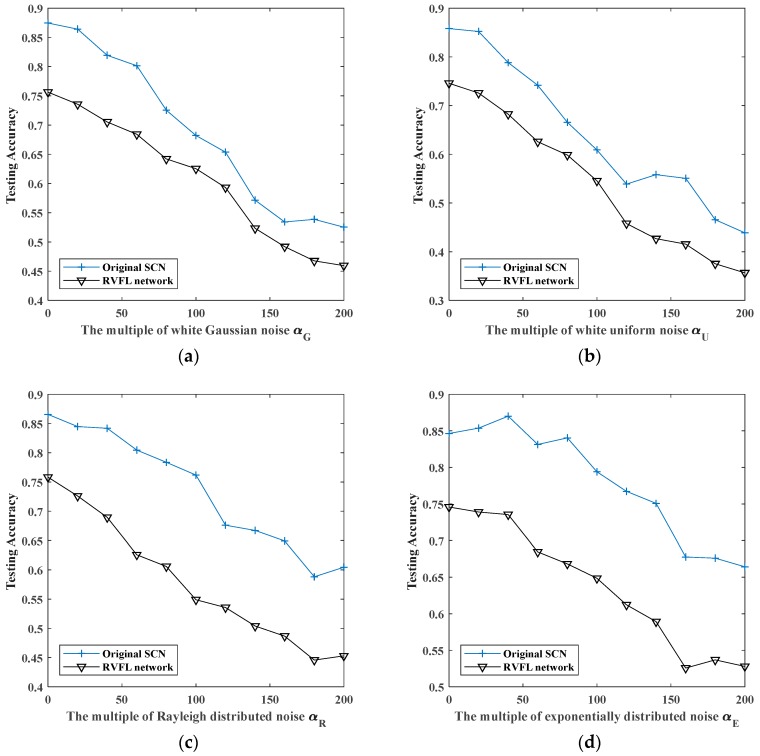
The maximum testing accuracy of original SCN and RVFL network with different noises: (**a**) white Gaussian noise; (**b**) white uniform noise; (**c**) Rayleigh distributed noise; (**d**) exponentially distributed noise.

Figure 15 shows the comparison of recognition effects of RVFL network and original SCN with different noises. We can see that the recognition effect of original SCN is 0.04–0.2 better than that of RVFL network, which is why we choose original SCN as the research object.

## 6. Discussion

**Figure 16:**
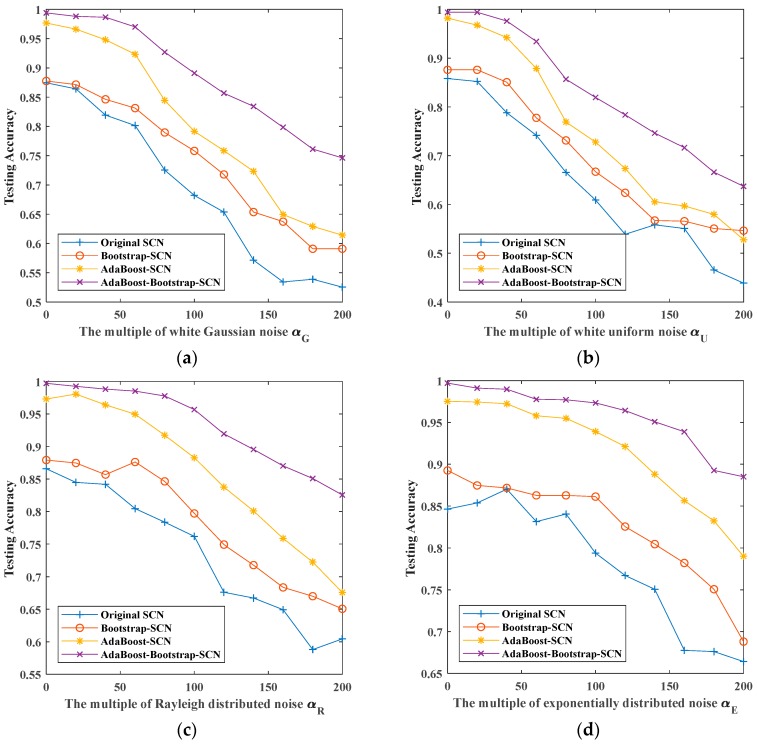
The maximum testing accuracy of different methods with different noises: (**a**) white Gaussian noise; (**b**) white uniform noise; (**c**) Rayleigh distributed noise; (**d**) exponentially distributed noise.

The recognition results of four kinds of noisy signals with four classifiers are shown in Figure 16 and Table 2. From the figures we can see that when there is no noise superimposed, the recognition rates of original SCN and Bootstrap-SCN are between 0.85–0.9, while the recognition rates of AdaBoost-SCN and AdaBoost-Bootstrap-SCN are greater than 0.95, which indicates that the use of AdaBoost can improve the recognition rate of the vibration signal in the case of noiseless superposition and noisy superposition. As the multiplier of superimposed noise increases, the recognition effect of each classifier decreases. However, the recognition effect of AdaBoost-Bootstrap-SCN is slower than other methods. When superimposing 200 times of noise, the recognition results of Bootstrap-SCN and AdaBoost-SCN have a small increase compared with that of original SCN, while the recognition results of AdaBoost-Bootstrap-SCN have a great improvement. Meanwhile, AdaBoost-Bootstrap-SCN is also improved by about 0.1 on the basis of AdaBoost-SCN when superimposing 200 times of noise.

It can be seen from the above discussion that: (1) after combining bootstrap sampling method with original SCN, the robustness of SCN can be improved slightly; (2) by combining AdaBoost method with SCN, the recognition rate of SCN can be improved over the entire noise range; (3) when bootstrap sampling method and AdaBoost method are combined with SCN simultaneously, AdaBoost-Bootstrap-SCN can improve the robustness of classifier on the basis of AdaBoost-SCN and achieve better recognition results.

## 7. Conclusions

In the case of small sample sets, this paper proposes three improved methods (Bootstrap-SCN, AdaBoost-SCN, and AdaBoost-Bootstrap-SCN) to improve the recognition rate of noisy optical fiber vibration signals. These methods enhance the robustness of SCN model by combining two ensemble method (Bootstrap and AdaBoost) when original SCN adds hidden layer nodes. These three improved methods have been trained and tested with noiseless and noisy optical fiber vibration signals. Compared with the existing research results, the prediction results of these methods are improved. In the noiseless vibration signal recognition task, the recognition accuracy is improved from 0.8612 of original SCN to 0.9955 of AdaBoost-Bootstrap-SCN. In the noisy vibration signal recognition task, the recognition accuracy is improved from 0.5582 (average) of original SCN to 0.7735 (average) of AdaBoost-Bootstrap-SCN. By combining SCN with ensemble learning methods, it can not only effectively reduce the impact of different noises on vibration signals, but also enhance the robustness of original SCN, which allows classifier to adapt to more complex and varied scenarios. Future efforts are aimed towards reducing the training time of AdaBoost-Bootstrap-SCN and improving the recognition effect of noisy optical fiber vibration signals.

## Figures and Tables

**Table 1:** Samples of training set and testing set

	Training Set	Testing Set	Overall
Pickaxe	167	42	209
Electric drill	192	48	240
Shovel	172	43	215
Overall	531	133	664

**Table 2:** Performance comparison of different classifiers

		RVFL Network	Original SCN	Bootstrap-SCN	AdaBoost-SCN	AdaBoost-Bootstrap-SCN
Original signals (average)		0.7516	0.8612	0.8813	0.9767	0.9955
Adding 200 times noise	White Gaussian noise	0.4596	0.5254	0.5910	0.6143	0.7463
	White uniform noise	0.3568	0.4388	0.5463	0.5278	0.6373
	Rayleigh distributed noise	0.4528	0.6045	0.6507	0.6759	0.8254
	Exponentially distributed noise	0.5279	0.6642	0.6881	0.7902	0.8850
	Average	0.4493	0.5582	0.6190	0.6520	0.7735

## References

[1] Qiu J., Liang W., Zhang L., Yu X., Zhang M. (2015). The early-warning model of equipment chain in gas pipeline based on DNN-HMM. J. Nat. Gas Sci. Eng..

[2] Lu W., Liang W., Zhang L., Liu W. (2016). A novel noise reduction method applied in negative pressure wave for pipeline leakage localization. Process. Saf. Environ. Prot..

[3] Allwood G., Wild G., Hinckley S. (2016). Optical Fiber Sensors in Physical Intrusion Detection Systems: A Review. IEEE Sensors J..

[4] Morteza Z., Mehdi S., Karim S. (2016). Pipeline leakage detection and isolation: An integrated approach of statistical and wavelet feature extraction with multi-layer perceptron neural network (MLPNN). J. Loss Prev. Process Ind..

[5] Liang S., Sheng X., Lou S., Feng Y., Zhang K. (2016). Combination of Phase-Sensitive OTDR and Michelson Interferometer for Nuisance Alarm Rate Reducing and Event Identification. IEEE Photon. J..

[6] Masoudi A., Newson T.P. (2016). Distributed optical fibre dynamic strain sensing. Rev. Sci. Instrum..

[7] Shang Y., Yang Y., Wang C., Liu X., Wang C., Peng G. (2016). Optical fiber distributed acoustic sensing based on the self-interference of Rayleigh backscattering. Measurement.

[8] Ukil A., Chen S., Andenna A. (2011). Detection of stator short circuit faults in three-phase induction motors using motor current zero crossing instants. Electr. Power Syst. Res..

[9] Marcum W.R., Cadell S.R., Ward T. (2015). The effect of jet location and duty cycle on the fluid mechanics of an unconfined free jet and its heat transfer on an impinging plate. Int. J. Heat Mass Transf..

[10] Yan J., Lu L. (2014). Improved Hilbert–Huang transform based weak signal detection methodology and its application on incipient fault diagnosis and ECG signal analysis. Signal Process..

[11] Bi F., Zheng T., Qu H., Pang L. (2016). A harmful-intrusion detection method based on background reconstruction and two-dimensional K-S test in an optical fiber pre-warning system. Photon. Sens..

[12] Qiu Z., Zheng T., Qu H., Pang L. (2016). A new detection method based on CFAR and DE for OFPS. Photon. Sens..

[13] Sun Q., Feng H., Li J., Jin S. Recognition of Pipeline Safety Events Applied to Optical Fiber Pre-warning System. Proceedings of the International Conference on Photonics, Optics and Laser Technology.

[14] Yen G.Y., Lin K.C. Wavelet Packet Feature Extraction for Vibration Monitoring. Proceedings of the 1999 IEEE International Conference on Control Applications.

[15] King D., Lyons W.B., Flanagan C., Lewis E. An Optical Fiber Water Sensor Utilizing Signal Processing Techniques and Artificial Neural Network Pattern Recognition. Proceedings of the Sensors.

[16] Makarenko A.V. Deep Learning Algorithms for Signal Recognition in long Perimeter Monitoring Distributed Fiber Optic Sensors. Proceedings of the 2016 IEEE 26th International Workshop on Machine Learning for Signal Processing (MLSP).

[17] Wang B., Pi S., Sun Q., Jia B. (2015). Improved wavelet packet classification algorithm for vibrational intrusions in distributed fiber-optic monitoring systems. Opt. Eng..

[18] Igelnik B., Pao Y.H. (1995). Stochastic choice of basis functions in adaptive function approximation and the functional-link net. IEEE Trans. Neural Netw..

[19] Wang D., Li M. (2017). Stochastic Configuration Networks: Fundamentals and Algorithms. IEEE Trans. Cybern..

[20] Smith R.G. (1972). Optical Power Handling Capacity of Low Loss Optical Fibers as Determined by Stimulated Raman and Brillouin Scattering. Appl. Opt..

[21] Sun B.N., Chang J., Lian J., Wang Z.L., Lv G.P., Liu X.Z., Wang W.J., Zhou S., Wei W., Jiang S. (2003). Accuracy improvement of Raman distributed temperature sensors based on eliminating Rayleigh noise impact. Opt. Commun..

[22] Candes E., Donoho D., Starck J.L., Murtagh F. (2003). Gray and color image contrast enhancement by the curvelet transform. IEEE Trans. Image Process..

[23] Qin Z., Bao X., Zhu T., Chen L. (2011). High Sensitivity Distributed Vibration Sensor Based on Polarization-Maintaining Configurations of Phase-OTDR. IEEE Photon. Technol. Lett..

[24] Liang Y., Lv L., Huang L., Wang D., Li P. (2018). Noise reduction method based on timed frequency hopping in long distance optical fiber sensing system. J. Comput. Methods Sci. Eng..

[25] Ölçer I., Öncü A. (2017). Adaptive Temporal Matched Filtering for Noise Suppression in Fiber Optic Distributed Acoustic Sensing. Sensors.

[26] Zhou Z.H. (2012). Ensemble Methods: Foundations and Algorithms.

[27] Zhang P.B., Yang Z.X. (2016). A novel AdaBoost framework with robust threshold and structural optimization. IEEE Trans. Cybern..

[28] Fernandes A., Utkin A., Eiras-Dias J., Silvestre J., Cunha J., Melo-Pinto P. (2018). Assessment of grapevine variety discrimination using stem hyperspectral data and AdaBoost of random weight neural networks. Appl. Soft Comput..

[29] Asim K.M., Idris A., Iqbal T., Martínez-Álvarez F. (2018). Seismic indicators based earthquake predictor system using Genetic Programming and AdaBoost classification. Soil Dyn. Earthq. Eng..

[30] Suess S., Okujeni A., Van Der Linden S., Hostert P. (2017). Ensemble Learning from Synthetically Mixed Training Data for Quantifying Urban Land Cover With Support Vector Regression. IEEE J. Sel. Top. Appl. Earth Obs. Remote Sens..

[31] Akila S., Reddy U.S. (2018). Cost-sensitive Risk Induced Bayesian Inference Bagging (RIBIB) for credit card fraud detection. J. Comput. Sci..

[32] Ng W.Y., Zhou X.C., Tian X., Wang X.Z., Yeung D.S. (2017). Bagging–boosting-based semi-supervised multi-hashing with query-adaptive re-ranking. Neurocomputing.

[33] Freund Y.E., Schapire R. (1997). A Decision—Theoretic Generalization of On-Line Learning and an Application to Boosting. J. Comput. Syst. Sci..

[34] Zhou Z.H. (2012). Machine Learning.

